# Bio-Inspired Polarized Skylight-Based Navigation Sensors: A Review

**DOI:** 10.3390/s121114232

**Published:** 2012-10-24

**Authors:** Salmah B. Karman, S. Zaleha M. Diah, Ille C. Gebeshuber

**Affiliations:** 1 Institute of Microengineering and Nanoelectronics, Universiti Kebangsaan Malaysia, 43600 UKM Bangi, Selangor, Malaysia; E-Mails: szaleha@eng.ukm.my (S.Z.M.D.); gebeshuber@iap.tuwien.ac.at (I.C.G.); 2 Department of Biomedical Engineering, Faculty of Engineering, University of Malaya, 50603 Kuala Lumpur, Malaysia; 3 Institute of Applied Physics, Vienna University of Technology, Wiedner Hauptstrasse 8-10/134, 1040 Vienna, Austria

**Keywords:** bioinstrumentation, bio-inspired, polarized skylight navigation sensor

## Abstract

Animal senses cover a broad range of signal types and signal bandwidths and have inspired various sensors and bioinstrumentation devices for biological and medical applications. Insects, such as desert ants and honeybees, for example, utilize polarized skylight pattern-based information in their navigation activities. They reliably return to their nests and hives from places many kilometers away. The insect navigation system involves the dorsal rim area in their compound eyes and the corresponding polarization sensitive neurons in the brain. The dorsal rim area is equipped with photoreceptors, which have orthogonally arranged small hair-like structures termed microvilli. These are the specialized sensors for the detection of polarized skylight patterns (e-vector orientation). Various research groups have been working on the development of novel navigation systems inspired by polarized skylight-based navigation in animals. Their major contributions are critically reviewed. One focus of current research activities is on imitating the integration path mechanism in desert ants. The potential for simple, high performance miniaturized bioinstrumentation that can assist people in navigation will be explored.

## 1. Introduction

Human and animals navigate for various needs—for finding food, for social reasons, for communication and others. Current navigation devices are mostly dependent on the global navigation satellite system (GNSS), the more fully operational system for global positioning compared to others. The variability of function and integration of the new generation of GNSS has increased the market demand for related products [1]. However its applications may be limited by the low precision of the signal under certain conditions such as in urban areas and in situations of intermittent coverage. Furthermore, the system is always at risk of being shut down during a conflict. A new system should be developed to overcome these limitations.

The development of a GPS-independent navigation system has been inspired by the skylight-based navigation employed by insects. These insects, with their tiny eyes and brains, are capable of navigating distances of hundred of meters (walking insects) [2] or many kilometers (flying insects) by utilizing the pattern of polarized skylight. Through replicating such insect navigation systems, it may be possible to provide a kind of “*navigational sense*” to people. The development of a bio-inspired polarized skylight navigation sensor that can expand the human sensory ability towards such a “*navigational sense*” necessitates the use of highly interdisciplinary bioinstrumentation. In order for this to operate effectively, there is a need to connect external devices to the human body. To connect the system with the human body, miniaturization of the devices needs to be performed. In this article existing bio-inspired polarized skylight-based navigation sensors are reviewed with the intention of examining whether it is possible to upgrade respective devices to miniaturized bioinstrumentation systems that can be utilized for human navigation, potentially in combination with established GPS-based systems, to overcome limitations of both approaches. Both through the development of GPS-independent navigation systems and improvements to current GPS systems bioinspiration of polarized skylight navigation sensors has already yielded tangible results. These devices have been tested for use as a navigational compass that can guide a mobile robot [3,4].

Lambrinos *et al.*[5] invented a GPS independent polarization compass model that mimics the principle of the desert ant navigation system. Chu and co-workers enhanced this polarization compass principle [4,6–9] and improved the error measurement. Further groups working in this field are Gao and Fan and their co-workers [10–16]. Lu and co-workers implemented polarized skylight detection mechanisms into existing GPS systems [17–19]. Fan *et al.*[10] implemented a new integrated navigation solution with polarized skylight with geomagnetism and GPS. Advanced miniaturized devices can then be linked to the human body (mainly *ex corpore* to avoid ethical issues) in order to expand the human sensory perception towards a polarized skylight-based “*navigational sense*”. This sensor would be very beneficial to humans, especially those who suffer from difficulties with their sight, are wheelchair bound or suffer from Parkinson's disease.

## 2. Polarized Skylight-Based Navigation in Nature

### 2.1. Navigation by Insects

The polarized skylight detecting ability that is inherent in many animals is an example of natural systems or processes that could be used as an inspiration for the development of a bioinstrumentation for a human “*navigational sense*”. Many animals are capable of traveling both long and short distances before using their natural navigation abilities to return to a given location [11]. Insects that navigate short distances, normally for essential daily activities such as locating food sources, include desert ants, honey bees, dung beetles and crickets; while animals and insects that use in-built navigational systems over long distances, normally seasonally for breeding or migratory purposes, include species such as sea turtles, migratory birds and pigeons and monarch butterflies. Insects use information cues to guide their navigation and one source of these is polarized skylight, which they utilize for the purposes of navigation and orientation [11]. Examples of insects that use polarized skylight in this manner include crickets [12], locusts [13], flies [14] and dung beetles [15]. The majority of members of the bee species are diurnal, while some are nocturnal [16]. Central place foragers such as bees and ants use a natural polarization compass both to measure and to adjust their traveling direction in the context of path integration [17–19]. Long-range migrators, such as locusts [20] and some lepidoptera, including monarch butterflies, are also believed to orient themselves by exploiting skylight polarization during their journeys [21]. However, this does remain a subject of debate, as in an alternative behavioral study monarch butterflies failed to respond to e-vector orientation [22]. Desert ants and honeybees use three types of information to navigate. The first of which is based on a path integration memory of the position of the food site with respect to the nest. Path integration is based on egocentric information and enables foraging ants to return to the nest from any position at any time using the shortest direct track without help of terrestrial cues such as landmarks or panoramic views [23–25]. The second type of information used to navigate is based on visual snapshot memories of features that were viewed in the vicinity of or on the way to the food site. Thirdly, these insects also use local vector memories of the direction and length of habitual route segments. Landmark guidance is based on learning and memorizing the positions of terrestrial landmarks, such as bushes and trees, as well as the panorama and skyline along their route and enables the ants to relocate at precise earth-based absolute location [25–27]. The odometer process is handled differently by honeybees and ants: the honeybee uses an optic flow (pattern of apparent motion) during the flight experience, while the desert ant measures its travelling distance by using proprioreceptive cues (ability to sense stimuli regarding its own position, motion and equilibrium) [18]. E-vector information collection in crickets and ants is done by the polarization-sensitive photoreceptors in DRA and neurons in optic lobe [28,29].

### 2.2. Polarized Skylight Pattern—Signal Cues Required for Navigation

Direct light from the sun is unpolarized; it is the scattering of skylight in the atmosphere and the reflection of skylight on water or wet soil surfaces, rocks and vegetation that produces polarized reflected light [30]. Skylight is partially plane-polarized, *i.e.*, in any direction of the sky a particular orientation of the electric field (e-vector) of skylight dominates [31,32]. Malus and Strutt (Lord Rayleigh) in 1871 first discovered the polarization of the skylight. They provided a first explanation and mathematical description of celestial polarization. Subsequently, Chandrasekhar [33] established the full modern theory of this phenomenon. Two decades later, in the 1970s, Sekera [34] developed a computer program that was capable of describing both the direction and the degree of polarization [35]. The scattering of skylight within the Earth's atmosphere creates not only intensity and spectral gradients, but also polarization. Polarization can be described with two parameters: the orientation of the plane in which the electric vector or e-vector of skylight vibrates (*direction of polarization* or *e-vector orientation*), and the strength of this phenomenon (*degree of polarization*). The pattern of e-vector orientation is also recognized as a polarization pattern. The e-vector pattern in the sky uniformly changes its orientation by 15° per hour and marks the position of the pole of the daytime sky [36]. Because the skylight collecting powers of insect facet lenses are too small to catch enough quanta from even the brightest stars, they are not even able to see the stars [37,38]. Polarization sensitivity differs according to species: honeybees, desert ants and flies use UV-receptors [39–41], while crickets use blue receptors [41–43] in polarization vision [40].

### 2.3. Architecture of Insect Compound Eyes

**Figure 1.**
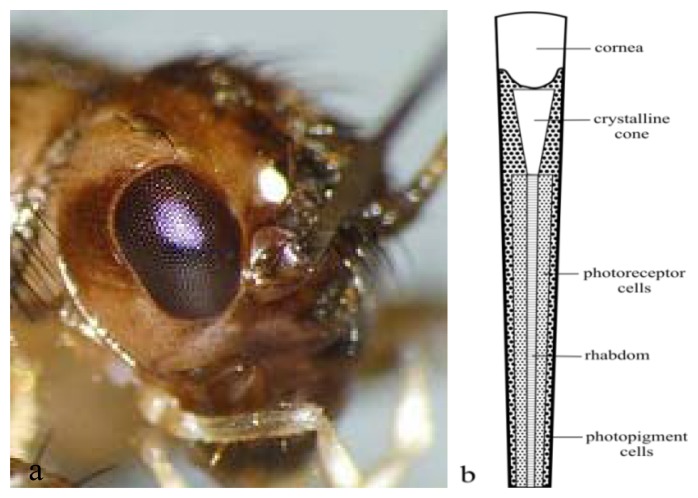
Structure of a compound eye. (**a**) Compound eyes of a crickets with permission by photographer (**b**) Details of a single ommatidium of a desert ant (adaptation from [58]) figure ©Zainalum.

Most arthropods, including insects and crustaceans, have a pair of compound eyes, either apposed (mostly insects active at day) or superposed (mostly insects active at night), that provide a wide field of vision. Compound eyes consist of three parts: a dorsal rim area (DRA), remaining dorsal area (DA) and a ventral area (VA). Figure 1(a) shows the compound eye of cricket. Each compound eye contains similar optical units, the ommatidia, of which the number varies in different insect species. There are up to 10,000 in dragonflies. Each ommatidium has its own cornea, lens and photoreceptor cells, which are used for distinguishing brightness and color. A single ommatidium guides skylight through a lens and cone into a channel called a rhabdom, which contains skylight-sensitive cells, as indicated in Figure 1(b). One ommatidium has one rhabdom. The rhabdom is the combined set of rhabdomeres. A rhabdomere is that part of the photoreceptor cell that consists of microvilli, which is a tube-like extension of the photoreceptor cell membrane. In this part of the cell membrane the rhodopsin molecules are embedded. Each rhodopsin has a characteristic absorption spectrum and thus determines the photoreceptor's sensitivity spectrum. When the rhabdomeres of the set of photoreceptors in one ommatidium are fused, the rhabdom is fused; when the rhabdomeres are spatially separated, the rhabdom is open. When the rhabdomeres of a fused rhabdom are organized in layers, the fused rhabdom is tiered. Rhabdom shapes and types are different according to the species. There are two types of rhabdoms: open (in flies, [44]) and fused (in bees and desert ants [45]). The fused rhabdom of butterflies is tiered [46,47]. The type of rhabdom directly impacts the shape of the spectral sensitivity curves of the respective photoreceptor cell [48]. In most arthropods and molluscs each ommatidium consists of retinula cells that are arranged in groups (seven photoreceptor cells in the dung beetle [15]; eight photoreceptor cells in flies [14]; nine photoreceptor cells in ants and honeybees [44]).

### 2.4. Ommatidia of the Dorsal Rim Area

Behavioral studies show that the detection of polarized skylight in insect eyes is mediated by the photoreceptors in ommatidia of the DRA (bees [49,50], ants [51], crickets [12]). Each ommatidium of the DRA contains two sets of homochromatic and highly polarization-sensitive photoreceptors with orthogonal microvilli (bee [45]; ants [52]; fly [41,53]; cricket [42,54,55]).

**Figure 2.**
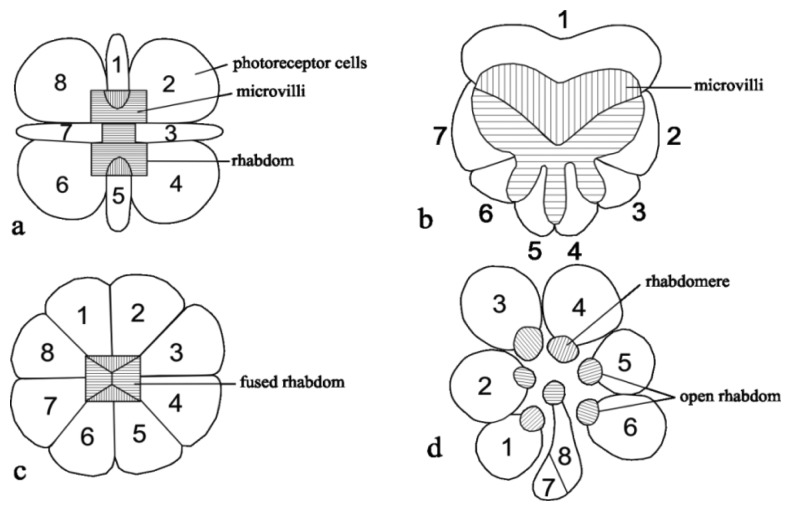
Cross-sections of ommatidia in the dorsal rim area (DRA). (**a**) The cross-section of an ommatidium of the desert ant *Cataglypis* with a dumb-bell shape and fused rhabdom. The microvilli of photoreceptor cells R1 and R5 are orthogonally to the remaining photoreceptor cells (figure adapted and modified from [58]). (**b**) Cross-section of a dung beetle ommatidium with a heart shape and fused rhabdom, microvilli R1 orthogonally with other photoreceptor cells (adaptation from [15]). (**c**) Cross-section of a honeybee ommatidium with rectangular shape and fused rhabdom. (**d**) Cross section of a fly with open rhabdom (adaptation from [44]) figure ©Zainalum.

The behavioral studies were confirmed through intracellular electrophysiological investigations that revealed that the photoreceptors in the DRA have much larger polarization sensitivity than in other parts of the eye (bees [45]; ants [52]; cricket [42]). In ants, *Cataglyphis*, the microvilli of DRA photoreceptors are aligned in parallel along the entire length of the cell, from the distal tip of the rhabdom down to its proximal end, near the basement membrane. The microvilli of the photoreceptor cells R1 and R5 (see Figure 2(a)) are always parallel to each other and perfectly perpendicular to the microvilli in the other photoreceptor cells [56]. Each ommatidium in the dorsal rim area has two photoreceptors, each of which is strongly sensitive to the e-vector orientation of plane-polarized skylight, with axes of polarization at right angles to one another. The axes of polarization direction of these ommatidia provide a fan-shaped orientation [57]. A cross-section of an ommatidium, a detailed rhabdom shape and the alignment of microvilli are shown in Figure 2.

### 2.5. Principles of Polarization Vision in Insects

In various insect species the detection of the oscillation plane of polarized skylight is mediated by a group of anatomically and physiologically specialized ommatidia that are situated in the DRA of the compound eye (see Section 2.4). Table 1 shows a mechanism of polarized skylight detection in various animals. In order to effectively use the information for the purposes of navigation, insects need to analyze the input signal of polarized skylight. This involves central processing stages, which include the lamina and medulla, the anterior lobe of the lobula, the anterior optic tract and tubercle, the lateral accessory lobe, and the central complex [59]. This process has been effectively imitated technologically in order to develop the polarization navigation sensor, which will be described in Section 3).

The mechanism for the detection of polarized skylight (e-vector detection) in the DRA involves the following situations:

- Optical axes that are always directed upwards. The visual field of the DRA has an elongated shape that extends from the upper front to the upper back, with the center directed somewhat to the contralateral side (bee [45]; desert ant [51,52]; cricket [42]).
- A rhabdom shape where the rhabdoms are shorter and have a larger cross-sectional area compared to other parts.
- Microvilli orientation, where the polarization-sensitive photoreceptors come in two sets that have their microvilli oriented at 90° to each other.
- Microvilli alignment: In the polarization-sensitive receptors, the microvilli are well aligned along the whole rhabdomere.
- Optics: Optical properties of the ommatidia are also affected [54].

During the central processing stage, the medulla polarization sensitive neurons act as integrators, while the central complex polarization neurons serve the function of a compass neuron that guides insect navigation [2]. The information of e-vector orientation is transferred in a form of sinus wave signal from the photoreceptor in the DRA to the central processing area. E-vector responses of particular photoreceptors in the DRA are pooled by a set of polarization neurons, which act as integrators (a set typically consists of three neurons). Each integrator is aligned to a particular e-vector tuning axis. The e-vector tuning axis varies according to the rotation movement of the insect body axis. The larger these variations are the more accurate is the neuron compass reading. The crossed-analyzer in insect eyes consists of two sets of e-vector analyzers, which are orthogonally arranged to each other, and are antagonistically responding to the e-vector. Each set of analyzer may consist of a polarization sensitive photoreceptor and a neuron [58].

The polarization sensitivity can be increased in insects through the application of several characteristics, including the orthogonal arrangement and high alignment of microvilli. The reduction in length prevents polarization reduction due to self-absorption. The increase in microvillar length favours sensitivity and enlarged the cross-section area. High polarization sensitivity in DRA photoreceptors and degraded optics skylight scattering structures in the cornea or missing screening pigment [18,54].

## 3. Insect-Inspired Polarized Skylight-Based Navigation Device

Thousands of years ago, the Vikings used skylight polarization to navigate across open oceans. Although much has been known regarding the Vikings' sunstones, there is a lack of concrete historical evidence on how the Vikings used such sunstones for navigation as well as their operation. In 1967, Ramskou, a Danish archaeologist, offered a suggestion on how the Vikings used polarized skylight when the position of the sun is behind clouds or fog, in which the sunstone is a polarizing crystal that is a transparent form of calcite, similar to the Icelandic spar as a linear polarizer [66]. In the “*Secrets of Viking Navigator*”, Karlsen [67] studied saga experiences and combined information with practice. The Vikings' navigators developed a unique instrument to determine the direction of the sun even in the presence of clouds and fog. Several sagas revealed that birefringent crystals called sunstones and a bearing board were implemented by the Vikings' navigators.

In the recent years, the development of the bio-inspired navigation sensor involved three different approaches and devices. The first approach was to use three pairs of photodetectors and linear film polarizers; the second involved the use of a camera-based polarization sensor; and the third employed a Division of Focal Plane (DoFP) polarimeter-based polarization sensor that was developed using complementary metal-oxide-semiconductor (CMOS) technology. These devices could be used as the basis for the development of new miniaturized bioinstrumentation devices to achieve a “*navigational sense*” in humans.

### 3.1. Polarization Navigation Sensor on Photodetector with Linear Film Polarizers

#### 3.1.1. Principle

**Figure 3.**
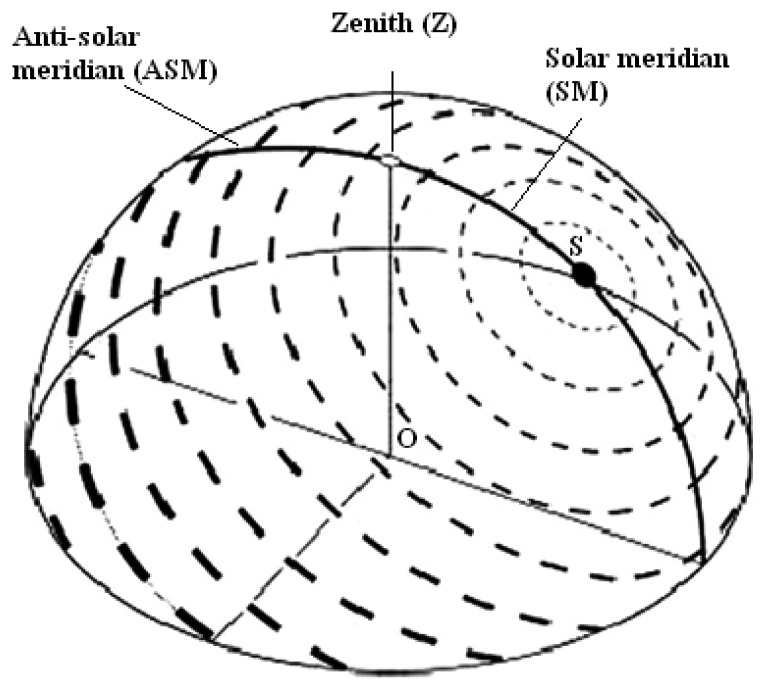
Three-dimensional representation of the pattern of polarization in the sky as experienced in point O. Orientation and width of the bars depict the direction and degree of polarization, respectively. A prominent property of the pattern is a symmetry line running through sun (S) and zenith (Z), called “solar meridian” (SM) on the side of the sun and “anti-solar meridian” (ASM) on the opposite side [51].

The calculation principles that are used in the sensor operation were based on the single scattering Rayleigh model [4]. In the single scattering Rayleigh model atmosphere, the direction of polarization is perpendicular to the plane of scattering determined by the observer, the celestial point observed and the sun (Figure 3) [4,5,9]. The working principle of the polarization navigation sensor was inspired by the desert ant, which utilizes polarization pattern as information cue to guide movement (see Sections 2.1 and 2.2). The polarization sensor was developed by mimicking the crossed-analyzer configuration that can be found in the polarization sensitive area of the desert ant eyes (see Section 2.5). The outputs of the polarization direction analyzers incorporate the sinusoidal dependence on the polarization angle (see Equation (1), explaining the features of polarization angle, polarized skylight intensity and polarization degree. As such, the current direction of the navigation sensor references the solar meridian [5,7].

#### 3.1.2. Components and Mechanism

The key components of the polarized skylight detection units of the navigation sensor consist of three pairs of polarization direction analyzers. Each polarization direction analyzer consists of a pair of polarization sensors (POL-sensors) and a log-ratio amplifier, which was inspired by the desert ant polarized skylight sensitive photoreceptor and polarization neuron [4–7]. Each POL-sensor consists of two pairs of photodetectors and linear film polarizers. The polarizing axis of the POL-sensors of each polarization direction analyzer were adjusted 90° to each other in order to produce the crossed-analyzer configuration, which serves to enhance the e-vector contrast sensitivity [5]. This configuration mimics the biological photoreceptor arrangement within each ommatidium of the DRA. The polarization skylight detection unit of the polarization navigation sensor was developed by mounting the three polarization direction analyzers on the base plate of the navigation sensor and adjusting them in such a manner that the polarizing axis of the positive channel was 0, 60, and 120 degrees with respect to the 0 degree reference of the sensor [5,7]. In the mobile robot application, the 0 degree reference of the sensor was with respect to the robot's body axis (mimicking the three types of cricket POL-neurons that are tuned to different e-vector orientation: 10°, 60°, 130°).

**Figure 4.**
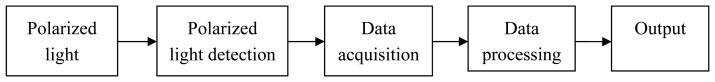
Simple block diagram of polarization navigation sensor [4].

As developed by Chu and co-workers [4] (Figure 4), the main parts of the bio-inspired polarization navigation sensor are the polarized skylight detection unit, data acquisition, data processing and connector to the output or another machine. In Chu's design [7], the six uniform arrangements of regular triangular prisms of the three polarization direction analyzers produce the hexagonal shape of the polarized detection unit, which is required for the device to function. Mounting the polarization navigation sensor on the mobile robot yielded the polarization compass. The block diagram of the polarization navigation sensor is depicted in Figure 4. The polarized light detection unit was mounted on the base plate, which incorporated the electronic parts. The amplifier and filter circuit of the polarization navigation sensor was designed by adopting 16 bits Analog-to-Digital Converter MAX195 in data acquisition. The center processor is an ARM7TDMI-based high performance 32-bit RISC microcontroller.

#### 3.1.3. Compensation of the System's Weaknesses

The integration of the sensor with further additional devices or algorithms was performed in order to compensate the weakness of the compass. These are additional ambient light, ocelli integration, a fuzzy logic controller algorithm and the development of an eye model. The ambient light sensor is used to resolve the ambiguity, since the sinusoidal function has a period of π, there will be two output angle values ø (*i.e.*, ø and ø + π) in each polarization direction analyzer. In Lambrinos's design [5], the eight ambient light sensors are arranged in two half circles that cover a visual field of 180 degree each. A rough estimate of the robot's heading with respect to the sun is obtained from the values from the ambient-light sensors. If the robot is oriented towards the sun, the ambient-light sensor with the visual field enclosing the solar meridian will have a stronger response than the other sensors. Chu *et al.*[4] improved this design by using only six ambient light sensors to solve the ambiguity. Each ambient light sensor consisted of standard photoresistors with a blue filter placed in front. It was directed horizontally with a visual field of approximately 60 degrees.

By mimicking the dragonfly, Chahl and Mizutani [68] integrated the ocelli and polarization sensors to provide improved compass headings and precise control of roll angle by stabilizing the sensor in the rolling direction of the robot. The polarization sensing elements use the optics to capture skylight from a region of the sky, this is then analyzed using a polarization sensor, which converts the skylight to voltage (see Section 3.1.2). The ocelli are simple eyes that are mounted on the front of the head (the head of the dragonfly). Before Chahl and Mizutani tested their sensor on the robot, they tested it in a flight test device that was equipped with ocelli to observe the stabilization quality of the sensor in pitch and roll [68]. The combination of lateral and longitudinal optical sensors allows full stabilization of the sensor in pitch and roll (in an ideal environment).

The algorithm of the fuzzy logic controller [8] was programmed in order to obtain robust performance and increase the efficiency and accuracy of the polarization navigation compass. The algorithm serves to direct the mobile robot to follow the trajectory in a smooth and continuous manner at the best possible precision. Through a fuzzy logic controller, the information obtained from the polarization sensor is mapped to the robot's velocity.

Nature's design of the compound eye is important in order to capture the image at an optimal level [69]. In order to mimic the design of compound eyes, a number of scientists have developed the eye model. Chu *et al.*[4] developed a cylinder shaped polarized detection unit that mimics the eyes of desert ants. Six cylinders were assembled in a uniform arrangement of three round-holed polarization direction analyzers with the blue filter on the top. The ratio of the radius and the height of the round holes 0.5, and they achieved a view field of approximately 53 degrees. The spectral sensitivity of the polarization channels ranged from approximately 400–520 nm with a maximum in the blue (460 nm). Gao and co-workers [70] developed a model of the desert ants eye by arranging a monocular eye structure in a 6 × 6 curved array: their monocular eye model consisted of a cylinder shape tube that contained two sets of polarizers and photodetectors. The first set consisted of a primary polarizer that was circular in shape and polarized the light that came from the light source. The second set was a secondary polarizer, which was constructed using a uniform arrangement of 6 small radius polarizers that were positioned at different angles of the polarizing axis.

### 3.2. Camera-Based Polarization Sensor

In 2001 Usher [71] used a normal camera with an external linear polarization filter to take two images, the second image was taken with the polarization filter set orthogonal with respect to its position in the first image. The camera used in this experimental investigation was the SONY XC-EU50CE camera. The blue component of the camera's RGB output was used for analysis, as polarization of sunlight is most apparent at ultraviolet and blue wavelengths (350–450 nm). Linear polarizing film was used as a filter to extract the polarized images. All images were smoothed using a two-dimensional Gaussian function. A mean intensity function as a function of polarization angle was derived from the two images and was comparable to the form proposed by Lambrinos [5].

Carey and Sturzl [72] developed the insect-inspired low-resolution omnidirectional vision system by incorporating a near-UV camera and a linear polarizer. By incorporating an additional RGB camera, the full range of the insect's visual spectrum was obtained, making it possible to capture and investigate the visual cues that insects use in flight control and navigation. This investigation could enhance the understanding of the insect navigation system and lead to the incorporation of a similar system in an autonomous mobile robot. The equipment employed was the SONY camera mentioned above, a video signal digitizing device and a UV transmitting glass filter to maximize UV photoreception at about 340 nm.

### 3.3. Division of Focal Plane (DOFP) Polarimeter-Based CMOS Polarization Imaging Sensor

#### 3.3.1. Principle

A polarization imaging sensor can also be developed by integrating a Division of Focal Plane (DoFP)-based polarimeter using a CMOS technique. Various groups have worked on the development of CMOS-based polarization imaging sensors [73–80]. The principle of this device is elaborated in detail in Andreou and Kalayjian [81]. State-of-the-art CMOS polarization image sensors consist of an array of integrated photodetectors and a micropolarizer, which measures polarization information in real time [79]. The ant rhabdomere structure served as inspiration for the development of the multiaxis micropolarizer [82].

Many scientists have focused on the development of sensors and improved fabrication techniques for micropolarizers. The current generation of these sensors is produced via micro- and nanofabrication techniques, which yield high-resolution polarization imaging sensors. The two types of polarization sensor that were developed are either based on a polymer film micropolarizer or on a metal wire grid micropolarizer.

The polymer film-based CMOS polarization imaging sensor using DoFP architecture was developed by Zhao *et al.*[73] and Gruev *et al.*[77]. Zhao *et al.* patterned the polymer film micropolarizer on a spin-coated azo-dye-1 film, where the micropolarizer fabricates in four directions of polarization; 0°, 90°, 45° and −45°. Gruev *et al.* developed a CMOS-based polarization-imaging sensor with dual tier polymer film micropolarizer in two different orientations, which are offset by 45°.

The main disadvantages of polymer polarizer-based polarization imaging sensors are the complicated fabrication processes—this has led to the creation of the metallic wire grid micropolarizer-based sensor [76]. Tokuda *et al.* developed the first generation of metallic wire grid polarizers of CMOS-based polarization imaging sensors [74,75,83]. Their sensor possesses a metal wire grid polarizer with a pitch size of 1,200 nm, with 1,880 × 1,880 μm chip size. This sensor was developed for sensing applications in microfluidics. The application of a CMOS-based metal wire grid micropolarizer for navigation compass was reported by Gruev *et al.*[76,78] and Sarkar *et al.*[79,80]. As described by Sarkar *et al.*[79], the variations of polarization information in real time with changes in the angular position of the incoming polarized skylight ray are shown to work as an effective compass.

#### 3.3.2. Key Components

The basic component of CMOS-based polarization imaging sensor is a DoFP architecture polarimeter, which combines imaging CCD elements and micropolarization filters on the same substrate [76]. The sensor consists of an array of these polarimeters, in a certain number of pixels, pixel sizes and dimensions, and micropolarizer types, dependent of the project requirement (Table 2). For example, Sarkar *et al.*[79] developed a sensor with 128 × 128 pixels, which occupies the total chip area of 5 × 4 mm^2^, fabricated using a 180 nm CMOS CIS process. The sensor has an embedded linear wire grid polarizer in each pixel, realized with the first metal layer of the process on top of a pinned photodiode (p+/n-/p-sub). The linear wire grid polarizer is a thin metal strips with a line/space of 240/240 nm (pitch of 480 nm). As in Sarkar *et al.*[79], the chip is divided into four main blocks, where the first block consists of the pixel array for signal capturing and processing, the second consists the analog readout circuit, the third block consists of the digital readout circuit, while the fourth block consists of a 7-bit counter and a column shift register.

**Figure 5.**
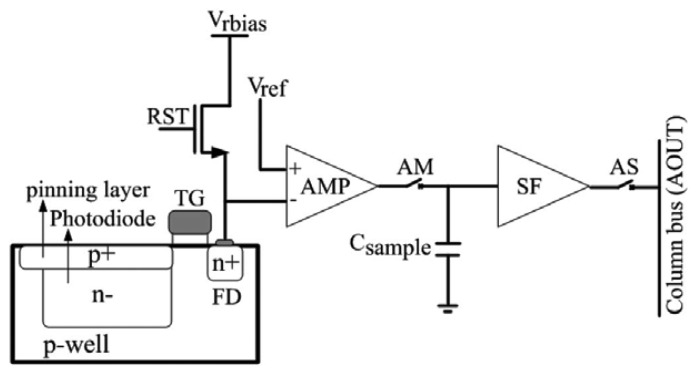
Simplified pixel architecture [80] (V_rbias_: Voltage reverse bias for reset transistor, V_ref_: Voltage reference for Buffer amplifier, RST: Reset transistor, TG: Transfer gate, FD: Floating diffusion, AMP: Buffer amplifier, AM: switch, C_sample_: Sampling capacitor, SF: Source follower, AS: switch).

The sensor pixels in the signal capturing block was split into three regions: Region 1 is used for normal imaging application, Region 2 is used for the detection of polarization with the polarizing angle of 0°, and 90° [79] and Region 3 is used for the detection of polarization with the polarizing angle of 0°, 45° and 90°. Figure 5 shows the simplified pixel architecture of the CMOS-based polarization imaging sensor using DoFP architecture. The image capture begins with a reset of the pixel by switch on the RST [80]. The voltage at the floating diffusion node FD is then set to the reset voltage Vrbias. The reset is global in nature for the entire pixel array. After the reset, the photodiode starts accumulating the photogenerated charge. The time spent accumulating the charge is referred to as the integration time or the exposure period. At the end of the integration time, the accumulated charge is transferred to FD.

#### 3.3.3. Design Improvement

To obtain the high performance of DoFP-based CMOS polarization image sensor, the size of the imaging array [77] and the extinction ratios (ER) [81] should be enlarged, while the size of the sensor pixel should be reduced [84]. The ER is greatly influenced by the structure of the micropolarizer filter. Reducing the thickness of the micropolarizer array layer could serve to increase the ER. The ER could also be improved by using high conductivity metal [85]. The pitch size of the micropolarizer has an effect on detecting the wavelength [80]. Smaller pitch size leads to higher optical performance [86]. For an EM wave to be absorbed by a wire grid, its wavelength should be larger than the pitch of the wire grid. The wavelength range of the visible spectrum is from about 300 to 720 nm; thus, a wire grid pitch should be less than 300 nm [80]. A smaller amount of micropolarizer polarization direction is required to reduce the sensor size and simplify the fabrication process [77]. The metallic wire grid polarizer can be used to detect the polarization pattern [79,80], since it has a function of selectively transmitting wavelengths [87]. The pitch size and ER of the several polarization sensors used for the navigation compass are shown in Table 2. Further projects that incorporate wire grid micropolarizer development will be described in the next section.

#### 3.3.4. Wire Grid Micropolarizer for a Polarized Skylight-Based Nano Size Navigation Sensor

**Figure 6.**
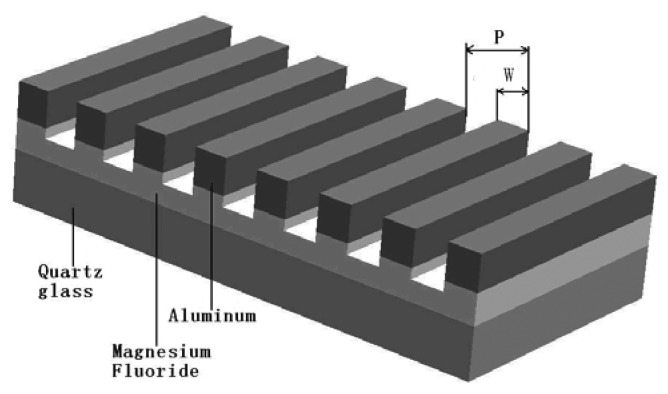
The structure of the designed wire grid grating polarizer (P: grid period (pitch), W: Grating width) [82].

The polarizer is an important component in a polarization direction analyzer [82]. The polarizers that are currently used in polarization navigation sensors, such as conventional birefringent crystal polarizers and the dichroic film polarizers, can't be used for nanoscale navigation sensors because of their size, features, the fact that they cannot be made based on miniaturization techniques and furthermore cannot be integrated with photoelectric capability. A wire grid micropolarizer is compact, has good polarization efficiency and exceptional reliability. The structure of the wire grid polarizer looks like mimicking the ant rhabdomere (Figure 6). As mentioned in Section 3.3.3, the pitch size has an effect to the performance of the polarization filter. Table 3 shows several designs of the wire grid micropolarizers with different ER, signal to noise ratio (SNR) and transmission efficiencies. The ER, SNR and transmission efficiencies of the wire grid micropolarizers are produced according to the different pitch size, sensor design and fabrication process of the wire grid micropolarizers.

Even though the development of some of the designs is for LCD applications [88,89], they also have the potential to be employed within the polarization navigation compass. Chen *et al.*[88] fabricated metallic wire grid polarizers through a nanoimprint, metal deposition and chemical mechanical polishing (CMP) process. The extinction ratio of the fabricated device was above 171.8 dB with a high transmittance of 91.6% at a wavelength of 650 nm of incident light. Suzuki *et al.*[89] successfully developed bilayered antireflection (AR) coatings on Al wire grid polarizers. FeSi_2_ as an absorptive material was deposited by the glancing angle deposition process on top of Aluminum wires that were covered with a SiO_2_ layer. Kim *et al.*[90] fabricated a nanowire polarizer that has the potential to be used as a wire grid micropolarizer. The device was fabricated using nanoimprint lithography (NIL), producing aluminum gratings of 200 nm period uniformly over an area of 3 × 3 cm^2^. This nanowire polarizer showed a polarization extinction ratio of 38 dB at a wavelength of 1,550 nm. The device demonstrates a cost-effective nanofabrication solution that has the potential to be applied to various devices with large-scale integration.

#### 3.3.5. Upgrading the Final Polarization image of DoFP-Based Polarization Sensor

The image from DoFP-based sensors is always degraded as a result of a number of problems. These include instantaneous-field-of-view (IFOV) errors, severe detector-to-detector nonuniformity signals and unresponsive or dead pixels [98]. To obtain a high-quality polarization image, the alignment, calibration and interpolation strategy of the sensors are critical. DoFP-based sensors present difficulties in alignment strategy since they require a mechanical process. The calibration of DoFP-based polarization sensors is discussed in detail by Tyo and Hayat, [99] and Tyo *et al.*[98]. Through calibration strategy, errors can be overcome via techniques such as nonuniformity correction and dead pixel replacement (DPR) [98,100–102]. There are two types of calibration techniques: radiometric and polarimetric [98,99]. The specialized polarimetric system calibration technique is discussed in detail in the works of Azzam *et al.*[103] and Goldstein and Chipman [104], who address Stokes vector simultaneous measurement and Fourier data reduction techniques respectively [98].

Since pixels with different polarization directions consist in a small neighborhood of the CMOS sensor, the estimations of Stokes vectors are obtained by combining measurements within this small neighborhood [105]. The implementation of this combined measurement may produce the estimated Stokes vector, which contains error and subsequently degrades the resulting polarization image. By interpolating the signal measurement from a defined neighborhood of pixels to a common reconstruction point, this error can be reduced. Several algorithms of interpolation have been developed, these are: bilinear, weight bilinear, bicubic, bicubic spline, approximated bicubic spline, bicubic convolution, gradient-based interpolation and correlation-based interpolation [106–108]. The bicubic spline interpolation method shows the best performance in reducing error and obtains higher modulation transfer function (MTF) gain compared to the bilinear, weight bilinear and bicubic interpolation methods; however, it does has computational complexities [106]. Gradient-based interpolation methods perform better than bicubic spline and bicubic convolution interpolation [107] while correlation-based interpolation performs better than the bicubic spline interpolation [108]. Gradient and correlation-based interpolation methods have the potential to significantly improve the CMOS-based polarization sensor.

## 4. Algorithms

### 4.1. Algorithm for Measurement and Analysis for Coupled Photodetector-Linear Film Polarizer-Based Polarization Sensor

The output of polarization sensor is described by the following equation [5]:

(1) $$S(Ø)=KI\left(1+\text{dcos}\left(2Ø-2{Ø}_{\text{max}}\right)\right)$$

where *I* is the total intensity given by *I* = *I_max_+ I_min_*.*I_max_* and *I_min_* being the maximum and minimum intensities, respectively. The degree of polarization is denoted by *d*, *Ø* is current orientation with respect to the solar meridian, *Ø_max_* is the value that maximizes *S(Ø)*, and *K* is a constant. The delogarithmized polarization sensor outputs of the direction 0°, 60° and 120° respectively are expressed by Equations (2–4):

(2) $${\tilde{p}}_{1}(Ø)=1-2{\bar{p}}_{1}(Ø)=d\cos(2Ø)$$

(3) $${\tilde{p}}_{2}(Ø)=1-2{\bar{p}}_{2}(Ø)=d\cos(2Ø-\frac{2\pi }{3})$$

(4) $${\tilde{p}}_{3}(Ø)=1-2{\bar{p}}_{3}(Ø)=d\cos(2Ø-\frac{4\pi }{3})$$

(5) $$d=\frac{1-2{\bar{p}}_{1}(Ø)}{\cos(2Ø)}$$

(6) $$Ø=\frac{1}{2}\text{arctan}\left(\frac{{\bar{p}}_{1}(Ø)+2{\bar{p}}_{2}(Ø)-\frac{3}{2}}{\sqrt{3}\left({\bar{p}}_{1}(Ø)-\frac{1}{2}\right)}\right)$$

The algorithm improvement is important for improving the polarization compass. From Equations (2–4), Zhao [9] eliminated the influence of the polarization degree, *d*, by presenting a new transform, where after simplification of *t_i_*, they got the value of output angle of the polarization sensor *S_i_* [Equation (10)] that was independent of *d*:

(7) $$t_{1}(Ø)=\frac{{\tilde{p}}_{1}(Ø)-{\tilde{p}}_{2}(Ø)}{\left|{\tilde{p}}_{1}(Ø)\right|+\left|{\tilde{p}}_{2}(Ø)\right|}$$

(8) $$t_{2}(Ø)=\frac{{\tilde{p}}_{2}(Ø)-{\tilde{p}}_{3}(Ø)}{\left|{\tilde{p}}_{2}(Ø)\right|+\left|{\tilde{p}}_{3}(Ø)\right|}$$

(9) $$t_{3}(Ø)=\frac{{\tilde{p}}_{3}(Ø)-{\tilde{p}}_{1}(Ø)}{\left|{\tilde{p}}_{3}(Ø)\right|+\left|{\tilde{p}}_{1}(Ø)\right|}$$

(10) $$S_{i}(Ø)=\frac{1}{2}\text{arctan}\left(\frac{t_{i}(Ø)}{\sqrt{3}}\right)$$

where *S_i_* (*I* = 1,2,3), *Ø_1_* = 0, *Ø_2_* = π/3, *Ø_3_* = 2π/3. The advantage of the new algorithm is its higher precision with identical input error due to the character of its piecewise function, even though the two algorithms have the same output function style.

The output of the polarization sensor could also be described by using the mathematical description known as Stokes parameters (shown in Equation (11)) [109]. Stokes parameters were first introduced by G.G. Stokes in 1852, who described the polarization state in four quantities. As shown in Equation (11), the four quantities: *S_0_*, *S_1_*, *S_2_* and *S_3_* could be assumed to be polarization values obtained from Filters 1, 2, 3 and 4 respectively. Here, the first filter is an isotropic filter, passing all states of polarization, Filter 2 is passing the linear state of polarization in the horizontal direction, while Filter 3 passes the linear state of polarization at a 45° direction. Filter 4 filters the circular polarization state only:

(11) $$\vec{S}=\left[\begin{array}{c} S_{0}\\ S_{1}\\ S_{2}\\ S_{3} \end{array}\right]$$

Filters of Type 1 and Type 2 are found in the works of Lambrinos *et al.* and Chu and co-workers, where the polarization states could be represented as Equations (12) and (13):

(12) $$S_{0}=I_{90{}^{\circ}}+I_{0{}^{\circ}}$$

(13) $$S_{1}=I_{90{}^{\circ}}-I_{0{}^{\circ}}$$

The theoretical basis for the system error model of the polarized-skylight angle measurement model (POLAMM) has been developed by Li *et al.*[110]. The system error model of POLAMM was derived to improve the calculation accuracy of the polarized light navigation sensor. Through this system error model, the system error source parameters could be recognized, and the system error could be compensated to a major extent. From Equation (1), the output of three POL sensors is shown as follows:

(14) $$S_{i}(Ø)=KI\left(1+\text{dcos}\left(2Ø-2{Ø}_{\max(i)}\right)\right)$$

where *S_i_* (*I* = 1,2,3), *Ø_1_* = 0, *Ø_2_* = π/3, *Ø_3_* = 2π/3.

According to the practical meaning, Equation (14) should satisfy (15):

(15) $$\{\begin{array}{l} -\frac{\pi }{2}<Ø\leq \frac{\pi }{2}\\ 0\leq d\leq 1 \end{array}$$

For convenience, *S_i_(Ø)* is denoted as *S_i_*. By substituting Equation (15) into Equation (14), Equation (16) is produced:

(16) $$\cos2Ø=\frac{S_{1}-\frac{S_{2}+S_{3}}{2}}{\sqrt{D}}$$

where:

(17) $$D=\frac{1}{2}{\left(S_{1}-S_{2}\right)}^{2}+\frac{1}{2}{\left(S_{2}-S_{3}\right)}^{2}+\frac{1}{2}{\left(S_{1}-S_{3}\right)}^{2}>0$$

By substituting Equation (17) into Equation (16), the equation of POLAMM could be obtained, as shown by Equation (18):

(18) $$Ø=\{\begin{array}{cc} \arcsin\left(\sqrt{\frac{1-co\text{s}2Ø}{2}}\right), & S_{2}\geq S_{3}\\ -\arcsin\left(\sqrt{\frac{1-\cos2Ø}{2}}\right), & S_{2}\geq S_{3} \end{array}$$

Here:

(19) $$\text{KId}=\frac{2\sqrt{D}}{3}$$

(20) $$d=\frac{2\sqrt{D}}{S_{1}+S_{2}+S_{3}}$$

From the differentiation method, the system error model of POLAMM is shown by:

(21) $$\Delta Ø=-\frac{1}{3d}\sum_{j=1}^{3}\text{sin}(2Ø-2{Ø}_{j})[1+\text{dcos}(2Ø-2{Ø}_{j})]\cdot \Delta K_{j}-\frac{2}{3}\sum_{j=1}^{3}sin^{2}(2Ø-2{Ø}_{j})\cdot \Delta {Ø}_{j}$$

### 4.2. Algorithm of CMOS-Based Sensor

The measurement in CMOS-based sensors is performed by calculating the Stokes parameters [89,94]. The electromagnetic radiation travel is utilized as input signal. The mathematical representation of an electromagnetic wave propagating in the *z* direction is given by Equation (22):

(22) $$E=E_{0}\text{cos}(kz-\omega t+\varphi_{0})$$

where *E_0_* is the amplitude, *k* is the wave constant (*k* = 2ð/ë), *ù* is the circular frequency (*ù* = *kc* = 2ð*c*/*ë*) and is the initial phase. As described in Section 4.1, the polarization state of an electromagnetic wave can be conveniently described using a set of parameters that are known as Stokes parameters (G.G. Stokes, 1852) [109]. These are represented in a column vector as in Equation (11), called as Stokes vector. Different with Lambrinos *et al.* and Chu and co-workers approach, this CMOS-based polarization imaging sensor is developed in three types of filters. By modifying the Stokes parameters described by G.G. Stokes, *S_0_*, *S_1_* and *S_2_* can be represented by Equations (23), (24), and (25) [80]:

(23) $$S_{0}=I_{90{}^{\circ}}+I_{0{}^{\circ}}$$

(24) $$S_{1}=I_{90{}^{\circ}}-I_{0{}^{\circ}}$$

(25) $$S_{2}=I_{45{}^{\circ}}-I_{0{}^{\circ}}$$

where *I_0°_* is the intensity of skylight after passing through a horizontal linear polarizer, *I_90°_* is the intensity after a vertical linear polarizer, and *I_45°_* is the intensity after a linear polarizer is placed at 45°. By calculating Equations (22) to (25), the degree of polarization, which is related to maximum and minimum transmitted intensity values, can be obtained.

### 4.3. Algorithm for Development of Ant Eye Model

Smith [111,112] developed an algorithm for a compass that was applied on robots and drones in light clouds. The working principle of this compass was inspired by the insect compound eye. The algorithm was created by measuring the position of the four points in the sky, where, *i.e.*, the angle χ between the polarized e-vector and the meridian equals ±π/4. The azimuth of these four points is invariant to variable cloud cover, provided that polarized skylight is still detectable below the clouds. The sum of these four azimuth values can be turned into a celestial compass, which is useful for the robot or drone. Compared with the photoreceptor-based design, a compass that uses this design offers a simpler device that offers more accuracy during navigation under cloudy sky.

## 5. Integration System

Lu *et al.*[113] introduced the polarized skylight integrated GPS-based navigation system in the three-dimensional world. The polarization measurement unit (POLMU) is a detection unit that consists of the mechanism and components of the polarization direction analyzer that was described in Section 3.1.2. By integrating the POLMU to the integrated GPS/INS navigation system, the attitude error correcting capabilities of the system was improved, producing better precision in the GPS/INS navigation system. Fan *et al.*[10] implemented a new integrated navigation solution with polarized skylight that assists with geomagnetism and GPS. The output of the analyzed polarization information is used as references in the measurement work of the integration system. By using a Kalman filter, the results of analyzed polarization information are combined with the results of the geomagnetism 3D compass to obtain the smallest error of angle results. By adjusting the results with GPS information, the final output results were obtained. The components of the polarization sensor are polarization direction analyzer, such as that described in Section 3.1.2, with the same mechanism.

## 6. Discussion

The insect navigation system, especially that of the desert ant, *Cataglyphis*, bees, locusts and crickets have offered useful insights into the development of polarization navigation sensors that have been utilized to assist the navigation of mobile robots (as described in Section 3). By further developing this robot polarization navigation sensor, it is possible that a new kind of human sense, a “*navigational sense*”, could be fabricated.

Insects, with their tiny eyes and brain, are capable of navigating over hundreds of meters through the utilization of the patterns of polarized skylight [2]. The dynamic properties of skylight polarization provide much useful information to any navigating animal and human utilizing specific devices. GNSS systems do not perform well under some conditions, and polarization based systems do not perform either under other conditions (e.g., indoors, night, artificial light). Both systems can be combined to bring the best of both worlds and cover the deficiencies of each other. The polarized skylight is appropriate to be used as the information in navigation activities because the predictor signal is simple with a static relationship between e-vector orientation and the sun's azimuth [36].

The qualitatively robust pattern of polarized skylight direction could be obtained under any condition and even in situations when the sun was not visible [114], such as under canopy and foliage [115], and during overcasts and heavy haze. This is because only a small section of clear sky is sufficient for the animals to obtain a compass bearing for accurate navigation [116]. The polarization angle pattern of this obscured sky is determined predominantly by scattering on cloud particles themselves [114]. Furthermore, the detection of polarization of downwelling skylight under clouds or canopies is most advantageous in the UV range, where the degree of polarization is lower than the threshold of polarization sensitivity in animals [2,117].

As described in this article, there are three major types of polarization navigation sensor designs that have been utilized in the robotic field: photodiode—linear film polarizer integrated-based design, camera and external polarizer-based design, and DoFP polarimeter-based CMOS sensor design. For the first design, the performance of the devices that were reported in this article is described as shown in Table 4. In the earlier project of this design, the error in the movement of the polarization navigation sensor mounted robot was approximately 13.5 cm, smaller than the error obtained in the latter project, which was 28 cm. However, the output angle in the latter project shows the smaller error than the earlier project, about ±1.3° in difference.

In the second design, the degree of polarization was not compensated. The polarization differential image or polarization summation image is the main task in the third design. This design also studied the image and the variation in the degree of polarization with respect to the orientation. In this design, the degree of polarization behavior with the orientation angle is evaluated. The degree of polarization information from this evaluation could be used to obtain the orientation angle for compass cue application. This third design offers a simpler computation calculation step and provides a system that is easier to integrate with other devices.

In order to develop a human “*navigational sense*”, polarized skylight sensor research in robotics can be applied to bioinstrumentation research through the use of miniaturization technology. In insects, the most sensitive photoreceptor within polarization detection is UV (bees, flies, ants) and blue photoreceptor (cricket) [43]. UV skylight is in a wavelength range from 330 to 350 nm [41,118], while blue skylight has a wavelength of about 450 nm [118]. As described in Section 3.3.3, for the wire grid micropolarizer to effectively detect the polarization signal, a wire grid pitch should be less than 300 nm. By applying the principle of the central processing stage of insect visual systems (see Section 2.5), the accuracy of the polarization navigation sensor could be increased by raising the number of the polarizing direction axes of the polarizer. Due to the small size and integration process, the multiple polarizing direction axes of the array of wire grid micropolarizers-based navigation sensor could only be developed using nanofabrication technology.

The extremely sensitive e-vector detection system used in crickets can be imitated through the development of a polarization navigation sensor for higher sensitivity. Crickets are active during daytime and at night. The crickets' threshold response possess at lower quantum flux induction than the threshold response by ants and honeybees. In crickets, the threshold response to the radiant quantum flux is about 2.5 × 10^7^ quanta cm^−2^·s^−1^ at 433 nm [43], where the effective quantum flux under the clear, moonless night sky (2 × 10^8^ quanta cm^−2^·s^−1^ at 380–500 nm) [119]. During daytime, the threshold for e-vector orientation of honey bees (*Apis mellifera*) for UV stimulus is about 10^10^–10^11^ quanta sm^−2^·s^−1^, much higher than that demonstrated by crickets.

## 7. Conclusions and Outlook·

The implementation of a bio-inspired polarized skylight navigation sensor that can expand the human sensory ability towards a “*navigational sense*” necessitates the creation of a connection between external devices and the human body. To enable this, miniaturization and integration of existing devices need to be performed, something that is possible using miniaturization technology. Through the application of miniaturization technology, the existing polarized skylight-based navigation sensor that is typically utilized for robot navigation could be enhanced for bioinstrumentation application by integrating them with various techniques, devices and system such as geo-informatics system (GIS) system. Health quality of patient that equipped with the polarization based “*navigational sense*” could be monitored by the central server at any time continuously or when requested, especially during their outdoor activities [120]. The central server, which is connected to at least three parties (patient, GIS and rescue center), will take first action if the patient's health quality decrease. For example, if the patient have heart problem, the patient's health monitoring device and “*navigational sense*” will send the health condition data and position data respectively to the central server. As the patient's health quality getting worse, the central server ready to send the ambulance to patient's location.

Weaknesses in the current engineering system when compared with the perfection of natural systems, were highlighted in this review. As such, new theories need to be developed that are capable of improving the approach. MEMS technology can potentially serve to create a system that can more accurately replicate the perfect natural systems with engineering systems or devices that have high functionality and intelligence. A bio-inspired polarized skylight-based MEMS navigation sensor would be very beneficial to humans, especially those who suffer from difficulties with their sight, are wheelchair bound or suffer from Parkinson's disease.

## Figures and Tables

**Table 1.** Mechanism of polarized skylight detections in various animals. RD–rhabdom, PR–photoreceptor, MV–microvilli.

**Animals**	**Organ**	**Mechanism**
Honey bees(*Apis mellifera*, *Apis cerana*) [54,60]	Compound eyes, ommatidia of the dorsal rim area (DRA)	RD: two populations of orthogonally arranged rhabdomeres; not twistedPR: UV receptor absorbing photopigments for polarization detection, maximum sensitivity for skylight polarized parallel to the microvilliMV: aligned in parallel along the length of each photoreceptor cell; microvilli orientation in a fan-like pattern
Desert ants(*Cataglypis bicolor, C. fortis)*[52,54,58,61]	Compound eyes, ommatidia of the DRA	RD: distal tips are dumb-bell shape and fused rhabdomPR: Polarization vision is mediated by UV receptor cells only; mutually perpendicular microvilliMV: aligned in parallel along the longitudinal axes of cells; microvilli orientation in a fan-like pattern
Cricket(*Gryllus campestris*) [18,58]	Compound eyes, ommatidia of the DRA	RD: fused and an elongated triangle rhabdom, contains two orthogonal microvilli orientationsPR: come in two sets that have their microvilli oriented perpendicularly oriented to each otherMV: strictly aligned along the rhadomeres
Beetle (*Scarabaeus zambesianus)*[15]	Compound eyes, ommatidia of the DRA	RD: heart-shaped with orthogonal microvilliPR: seven photoreceptor rhabdomeresMV: the microvilli of photoreceptor 1 are parallel but perpendicular to photoreceptor 2-7
Monarch butterfly(*Danaus plexippus)*[21,55]	Compound eyes, ommatidia of the DRA	RD: wide and short rhabdomsPR: two types of photoreceptor with mutually orthogonal microvilli orientation and well-aligned microvilli in each receptorMV: aligned in different planes to optimize skylight reception at all angles for more global photoreceptor activities
Butterflies(*Pieris rapae, Papilio crucivora, Colias erate*) [46,47]	Compound eyes, ommatidia	RD: fused rhabdomRhabdomere: rhabdomere consists of microvilli containing the rhodopsinPhotoreceptor: nine photoreceptors in three groups according to the position of their rhabdomere and specialized for polarization visionMV: microvilli contain rhodopsin
Flies (*Calliphora erythrocephala, Musca domestica, Drosophila melanogaster)*[14,41,62]	Compound eyes, ommatidia of the DRA	RD: open rhabdomPR: have eight photoreceptor cells, with six of them arranged in a trapezoidal pattern around the tiered rhadom and R7 and R8 specialized for detection of polarized skylight and high polarization sensitivityMV: orthogonally arranged
Spider(*Drassodes cupreus*) [63]	Secondary eyes, tapetum	Tapetum: acts as a polarizer, canoe-shaped tapeta; microvilli inside tapetumPR: sensitive to the plane of polarization of skylight, orthogonally arranged microvilli
Mantis shrimp*(Odontodactylus scyllarus)*[64]	Compound eyes, ommatidia of the DRA	Ommatidia: form 6 parallel rows, called midbandPR: specialized for UV (linearly polarized), for colour (blue-green) or polarization vision. Cells respond to skylight with an e-vector oriented parallel to the mid-band and with an e-vector oriented perpendicular to mid-band. Orthogonal arrangement of UV-sensitive photoreceptor cells; quarter-wave retarders.MV: parallel microvilli for polarization sensitivity
Locust *(Schistocerca gregaria)*[13,20]	Compound eyes, ommatidia of the DRA	RD: fused rhabdomPR: largely photoreceptors for blue with high polarization sensitivityMV: microvilli of photoreceptor cell 7 are oriented perpendicularly to microvilli of photoreceptors 1, 2, 5, 6 and 8; microvilli photoreceptor 3 and 4 are irregular; microvilli orientation are arranged in a fan-like pattern
Cephalopods (squid, cuttlefish and octopus) [64,65]	Complex skin with pigmented chromatophore organs and structural light reflectors (iridophores)	PR: detect linearly polarized skylight by reflectionMV: orthogonal arrangement of microvilliIridophores: contain stacks of protein plates interspersed by cytoplasm spaces, produce colorful linearly polarized reflective patterns

**Table 2.** Development progress of CMOS polarization imager.

**Project Author, Year**	**Polarizer on Chip (Pitch)–Metal Type**	**Pixel Size (μm^2^)**	**Pixel Number**	**Chip Size (μm^2^)**	**Extinction Ratio (ER)**	**SNR (dB)**	**Micropolarizer Type and Direction**	**Fabrication Process**
Tokuda, 2009 [75]	1,200 μm	20 × 20	30 × 30	1,880 × 1,880	2.03			0.35 mm 2 poly 4 metal standard CMOS
Zhao, 2009 [73]	10 μm -polymer				100		four directions of polarization; 0°, 90°, 45° and −45°	Spin coating and UV photo-lithography
Gruev, 2010 [76]	140 nm-Al		1,000 × 1,000		58	45	micropolarizers with four different orientations offset by 45°	
Perkin, 2010 [78]	130 nm-Al		1,000 × 1,000		58	45	four polarizer filter array (0°, 45°, 90°, 135°)	
Perkin, 2010 [78]	130 nm-Al		1,000 × 1000		58	45	two polarizer filter array (0°, 45°)	
Gruev, 2010 [77]	polymer	18 × 18	100 × 100		13	43.3	Dual tier polymer film with two different orientation offset by 45°	0.5 μm 2 poly 3 metal UMC CIS
Sarkar, 2010 [79,80]	480 nm	25 × 25	128 × 128	4,000 × 5,000	22	33	Combination of two types of micro-polarizer (first type: 2 direction of polarization; 0° and 90°; second: 3 direction of polarization; 0°, 45° and 90°)	0.18 μm 1 poly 3 metals UMC CIS

**Table 3.** Nano wire grid polarizers.

**Year, Ref.**	**Metal of Wire Grid**	**Grid Period (Pitch) (nm)**	**Extinction Ratio (ER)**	**SNR (dB)**	**Transmission Efficiencies (%)**

1998 [91]	Al	310–450	30		
2004 [90]	Al	200			38
2007 [82]	Al	200	>370		>61.5
2007 [92]	Al	118			85–90
2008 [86]	Al	80		47–70	38–94
2008 [93]		446			40
2010 [85]	Al	140			
2010 [94]		335			
2011 [95]	Al	200		>30	>75
2011 [96]	Al	350			
2011 [88]	Al	240	>171.8		91.6
2011 [89]	Al	150			
2012 [97]		300			

**Table 4.** Device performance.

**Group, Year, Reference**	**Output Angle Error**	**Robot Movement Error**	**Calibration Method**

Lambrinos, 2000 [5]	±1.5°	13.5 cm	Mobile robot (*Sahabot*) navigation
Chu, 2008 [4]	±0.2°		
Chu, 2009 [8]		28 cm	Mobile robot navigation

## Glossary

arthropods: animals lacking a backbone, with jointed limbs and a segmented body with an exoskeleton made of chitin.
*Cataglyphis*: a genus of ants
cephalopods: molluscs such as octopus, squid and cuttlefish
chromatophore: a cell or cell organelle that contains pigment
Coleoptera: beetles
cornea: transparent part in front of the eye
diurnal: active during the day
dorsal: the top, back or uppermost surface of an animal oriented with its head forward
egocentric: self-centred
*Gryllus*: a genus of crickets
Hemiptera: insects such as cicadas, aphids, planthoppers or shield bugs
homochromatic: containing or using only one colour
iridophore: a pigment cells that reflecting light
lamina: a thin layer, plate, or scale of sedimentary rock, organic tissue, or other material
Lepidoptera: insects such as butterflies and moths
Medulla: the inner region of an organ or tissue
microvilli: small ‘hairs’, component of insect photoreceptors
monochromatic: containing or using only one colour
ocellus: simple eye: an eye having a single lens
odometer (odograph): device that indicates the distance travelled
ommatidium: optical unit in compound eyes
proprioreceptive: ability to sense stimuli arising within the body regarding position, motion and equilibrium
protrusion: something that protrudes
rhabdom: light guide in insect compound eyes
rhabdomere: reflective inner border of rhabdom
tapetum: reflective layer in the eyes of many animals, causing them to shine in the dark
tubercle: small rounded projection or protuberance, esp. on a bone or on the surface of an animal or plant
ventral: of, on, or relating to the underside of an animal or plant

## References

[1] Mendizabal J., Berenguer R., Melendez J. (2009). GPS and Galileo: Dual RF Front-End Receiver Design, Fabrication, and Test.

[2] Wehner R. (2003). Desert ant navigation: How miniature brains solve complex tasks. J. Comp. Physiol. A.

[3] Shashar N., Sabbah S., Cronin T.W. (2004). Transmission of linearly polarized light in seawater: Implications for polarization signaling. J. Exp. Biol..

[4] Chu J., Zhao K., Zhang Q., Wang T. (2008). Construction and performance test of a novel polarization sensor for navigation. Sens. Actuators A: Phys..

[5] Lambrinos D., Möller R., Labhart T., Pfeifer R., Wehner R. (2000). A mobile robot employing insect strategies for navigation. Robot. Auton. Syst..

[6] Chu J., Zhao K., Wang T., Zhang Q. Research on a Novel Polarization Sensor for Navigation.

[7] Chu J., Zhao K., Zhang Q., Wang T. Design of a Novel Polarization Sensor for Navigation.

[8] Chu J., Wang H., Chen W., Li R. Application of a Novel Polarization Sensor to Mobile Robot Navigation.

[9] Zhao K., Chu J., Wang T., Zhang Q. (2009). A novel angle algorithm of polarization sensor for navigation. IEEE T. Instrum. Meas..

[10] Fan Z., Gao J., Pan D., Cui S. (2009). The implementation of a new integrated navigation solution with polarized-light assisting with geomagnetism and GPS. Geomatics Infor. Sci. Wuhan Univ..

[11] Rodrigo T. (2002). Navigational strategies and models. Psicológica.

[12] Brunner D., Labhart T. (1987). Behavioural evidence for polarization vision in crickets. Physiol. Entomol..

[13] Mappes M., Homberg U. (2004). Behavioral analysis of polarization vision in tethered flying locusts. J. Comp. Physiol. A.

[14] von Philipsborn A., Labhart T. (1990). A behavioral study of polarization vision in the fly. Musca domestica. J. Comp. Physiol. A.

[15] Dacke M., Nordström P., Scholtz C.H. (2003). Twilight orientation to polarised light in the crepuscular dung beetle. Scarabaeus zambesianus. J. Exp. Biol..

[16] Somanathan H., Kelber A., Borges R., Wallén R., Warrant E. (2009). Visual ecology of Indian Capenter bees II: Adaptations of eyes and ocelli to nocturnal and diurnal lifestyles. J. Comp. Physiol. A.

[17] Collett M., Collett T.S. (2000). How do insects use path integration for their navigation?. Biol. Cybern..

[18] Labhart T., Meyer E.P. (2002). Neural mechanisms in insect navigation: Polarization compass and odometer. Cur. Opin. Neurobiol..

[19] Wehner R., Sriinivasan M.V., Jeffery K.J. (2003). Path Integration in Insects. The Neurobiology of Spatial Behavior.

[20] Homberg U. (2004). In search of the sky compass in the insect brain. Naturwissenschaften.

[21] Reppert S.M., Zhu H., White R.H. (2004). Polarized light helps monarch butterflies navigate. Curr. Biol..

[22] Stalleicken J., Labhart T., Mouritsen H. (2005). Physiological characterization of the compound eye in monarch butterflies with focus on the dorsal rim area. J. Comp. Physiol. A.

[23] Kohler M., Wehner R. (2005). Idiosyncratic route-based memories in desert ants, *Melophorus bagoti* How do they interact with path-integration vectors?. Neurobiol. Learn. Mem..

[24] Narendra A. (2007). Homing strategies of the Australian desert ant *Melophorus bagoti*. II. Interaction of the path integrator with visual cue information. J. Exp. Biol..

[25] Graham P., Cheng K. (2009). Ants use the panoramic skyline as a visual cue during navigation. Curr. Biol..

[26] Wystrach A., Schwarz S., Schultheiss P., Beugnon G., Cheng K. (2011). Views, landmark and routes: how do desert ants negotiate an abstacle course?. J. Comp. Physiol. A.

[27] Graham P., Cheng K. (2009). Which portion of the natural panorama is used for view-based navigation in the Australian desert ant?. J. Comp. Physiol. A.

[28] Labhart T. (1988). Polarization-opponent interneurones in the insect visual system. Nature.

[29] Labhart T. (2000). Polarization-sensitive interneurons in the optic lobe of the desert ant. Cataglyphis bicolor. Naturwissenschaften.

[30] Wehner R. (1983). The Perception of Polarized Light. The Biology of Photoreceptor.

[31] Strutt J. (1871). On the light from the sky, its polarization and colour. Philos. Mag..

[32] Wehner R., Lehrer M. (1997). The Ant's Celestial Compass System: Spectral and Polarization Channels. Orientation and Communication In Arthropods.

[33] Chandrasekhar S. (1950). Radiative Transfer.

[34] Sekera Z. (1970). Reciprocity relations for diffuse reflection and transmission radiative transfer in planetary atmosphere. Astrophysics.

[35] Horváth G. (1995). How do water insects find their aquatic habitat?. Természet Világa Special Issue.

[36] Brines M.L. (1980). Dynamic patterns of skylight polarization as clock and compass. J. Theor. Biol..

[37] Rodieck R.W. (1973). The Vertebrate Retina. Principle of Structure and Function.

[38] Kirschfeld K. (1974). The absolute sensitivity of lens and compound eyes. Z. Noturforsch.

[39] Duelli P., Wehner R. (1973). The spectral sensitivity of polarized light orientation in *Cataglyphis bicolor* (Formicidae, Hymenoptera). J. Comp. Physiol..

[40] Labhart T. (1980). Specialized photoreceptors at the dorsal rim of the honeybee's compound eye-polarizational and angular sensitivity. J. Comp. Physiol..

[41] Hardie R.C. (1984). Properties of photoreceptor-R7 and photoreceptor-R8 in dorsal marginal ommatidia in the compound eyes of *Musca* and *Calliphora*. J. Comp. Physiol..

[42] Labhart T., Hodel B., Valenzuela I. (1984). The physiology of the cricket's compound eye with particular reference to the anatomically specialized dorsal rim area. J. Comp. Physiol..

[43] Herzmann D., Labhart T. (1989). Spectral sensitivity and absolute threshold of polarization vision in crickets: A behavioral study. J. Comp. Physiol. A.

[44] Menzel R., Snyder A.W., Hopper W.L., Markl H., Ziegler H. (1983). Photoreceptor Optics-Structure and Function of Photoreceptor.

[45] Labhart T. (1980). Specialized photoreceptors at the dorsal rim of the honeybee's compound eye—Polarizational and angular sensitivity. J. Comp. Physiol..

[46] Pirih P., Arikawa K., Stavenga D.G. (2010). An expanded set of photoreceptors in the Eastern Pale Clouded Yellow butterfly. Colias erate. J. Comp. Physiol. A.

[47] Stavenga D.G., Arikawa K. (2011). Photoreceptor spectral sensitivities of the Small White butterfly *Pieres rapae crucivora* interpreted with optical modelling. J. Comp. Physiol. A.

[48] Briscoe A.D., Chittka L. (2001). The evolution of color vision in insects. Annu. Rev. Entomol..

[49] Rossel S., Wehner R. (1984). Celestial orientation in bees: The use of spectral cues. J. Comp. Physiol. A.

[50] Wehner R., Strasser S. (1985). The POL area of the honey bee's eye: Behavioral evidence. Physiol. Entomol..

[51] Wehner R. (1982). Himmelsnavigation bei insekten neurophysiologie und verhalten. Neujahrsbl Naturforsch Ges Zürich.

[52] Labhart T. (1986). The electrophysiology of photoreceptors in different eye regions of the desert ant. Cataglyphi bicolor. J. Comp. Physiol. A.

[53] Hardie R.C., Ottoson D. (1985). Functional Organization of the Fly Retina. Progress in Sensory Physiology.

[54] Labhart T., Meyer E.P. (1999). Detectors for polarized skylight in insects: A survey of ommatidial specializations in the dorsal rim area of the compound eye. Microsc. Res. Tech..

[55] Labhart T., Baumann F., Bernard G.D. (2009). Specialized ommatidia of the polarization-sensitive dorsal rim area in the eye of monarch butterflies have non-functional reflecting tapeta. Cell Tissue Res..

[56] Meyer E.P., Domanico V. (1999). Microvillar orientation in the photoreceptors of the ant. Cataglyphis bicolor. Cell Tissue Res..

[57] Rossel S. (1993). Navigation by bees using polarized light. Comp. Biochem. Physio. A.

[58] Nilsson D.E., Labhart T., Meyer E.P. (1987). Photoreceptor design and optical properties affecting polarization sensitivity in ants and crickets. J. Comp. Physiol. A.

[59] Homberg U., Hofer S., Pfeiffer K., Gebhardt S. (2003). Organisation and neural connections of the anterior optic tubercle in the brain of the locust. Schistocerca gregaria. J. Comp. Neurol..

[60] Wehner R., Bernard G.D. (1980). Intracellular optical physiology II. Polarization sensitivity. J. Comp. Physiol..

[61] Mote M.I., Wehner R. (1980). Functional characteristics of photoreceptors in the compound eye and ocellus of the desert ant. Cataglyphis bicolor. J. Comp. Physiol. A.

[62] Wolf R., Gebhardt B., Gademann R., Heisenberg M. (1980). Polarization sensitivity of course control in. Drosophila melanogaster. J. Comp. Physiol. A.

[63] Dacke M., Doan T.A., O'carroll D.C. (2001). Polarized light detection in spiders. J. Exp. Biol..

[64] Cronin T.W., Shashar N., Caldwell R.L., Marshall J., Cheroske A.G., Chiou T. (2003). -H. Polarization vision and its role in biological signaling. Integr. Comp. Biol..

[65] Mäthger L.M., Shashar N., Hanlon R.T. (2009). Do The cephalopods communicate using polarized light reflections from their skin?. J. Exp. Biol..

[66] Ramskou T. (1967). Solstenen. Skalk.

[67] Karlsen L.K. (2003). Secrets of the Viking Navigators.

[68] Chahl J., Mizutani A. (2012). Biomimetic attitude and orientation sensors. IEEE Sens. J.

[69] Fermüller C., Aloimonos Y. (1998). Ambiguity in structure from motion: Sphere *versus* plane. Int. J. Comput. Vision.

[70] Feng W., Gao J., Ren S., Wu K. (2008). Modeling and simulation of *Cataglyphis* compound eye. Chin. J. Scientific Instrum..

[71] Usher K., Ridley P., Corke P. A Camera as a Polarized Light Compass: Preliminary Experiments.

[72] Carey N., Sturzl W. An Insect-Inspired Omnidirectional Vision System Including UV-Sensitivity and Polarisation.

[73] Xiaojin Z., Boussaid F., Bermak A., Chigrinov V.G. (2009). Thin photo-patterned micropolarizer array for CMOS image sensors. IEEE Photon. Tech. L.

[74] Tokuda T., Yamada H., Sasagawa K., Ohta J. Polarization-Analyzing Image Sensor Based on Standard CMOS Technology.

[75] Tokuda T., Yamada H., Sasagawa K., Ohta J. Polarization-analyzing CMOS Image Sensor Using Monolithically Embedded Polarizer for Microchemistry Systems.

[76] Gruev V., Perkins R., York T. (2010). CCD polarization imaging sensor with aluminum nanowire optical filters. Opt. Express.

[77] Gruev V., Van der Spiegel J., Engheta N. (2010). Dual-tier thin film polymer polarization imaging sensor. Opt. Express.

[78] Perkins R., Gruev V. (2010). Signal-to-noise analysis of Stokes parameters in division of focal plane polarimeters. Opt. Express.

[79] Sarkar M., San Segundo Bello D., van Hoof C., Theuwissen A. Integrated Polarization Analyzing CMOS Image Sensor for Autonomus Navigation Using Polarized Light.

[80] Sarkar M., San Segundo Bello D., Van Hoof C., Theuwissen A.J.P. (2011). Integrated polarization-analyzing CMOS image sensor for detecting the incoming light ray direction. IEEE Trans. Instrum. Meas..

[81] Andreou A.G., Kalayjian Z.K. (2002). Polarization imaging: Principles and integrated polarimeters. IEEE Sens. J..

[82] Meng F., Chu J., Han Z., Zhao K. The Design of the Sub-Wavelength Wire-Grid Polarizer.

[83] Tokuda T., Yamada H., Sasagawa K., Ohta J. (2009). Polarization-analyzing CMOS image sensor with monolithically embedded polarizer for microchemistry systems. IEEE Trans. Biomed. Circ. S..

[84] Momeni M., Titus A.H. (2006). An analog VLSI chip emulating polarization vision of octopus retina. IEEE Trans. Neural Netw..

[85] Takano K., Morimoto I., Yokoyama H., Hangyo M. Wire-Grid Polarizer in the Terahertz Region Fabricated by Nanoimprint Technology.

[86] Yang Z.Y., Zhao M., Dai N.L., Yang G., Long H., Li Y.H., Lu P.X. (2008). Broadband polarizers using dual-layer metallic nanowire grids. IEEE Photonics Technol. Lett..

[87] Hansen D., Gardner E., Perkins R. (2002). The display applications and physics of the ProFlux™ wire grid polarizer. SID Digest.

[88] Chen C.M., An T.P., Hung Y.M., Sung C.K. (2011). Fabricating insertion structures for metallic wire grid polarizers by nanoimprint and CMP process. Microelectron. Eng..

[89] Suzuki M., Takada A., Yamada T., Hayasaka T., Sasaki K., Takahashi E., Kumagai S. (2011). Antireflection coatings with FeSi_2_ layer: Application to low-reflectivity wire grid polarizers. Thin Solid Films.

[90] Kim J.S., Lee K.D., Ahn S.W., Kim S.H., Park J.D., Lee S.E., Yoon S.S. (2004). Fabrication of nanowire polarizer by using nanoimprint lithography. J. Korean Phys. Soc..

[91] Doumuki T., Tamada H. An Aluminum-Wire Grid Polarizer Fabricated onto a Gallium Arsenide Photodiode.

[92] Wang J., Walters F., Liu X., Sciortino P., Deng X. (2007). High-performance, large area, deep ultraviolet to infrared polarizers based on 40 nm line/78 nm space nanowire grids. Appl. Phys. Lett..

[93] Yoon Y.T., Lee H.S., Lee S.S., Kim S.H., Park J.D., Lee K.D. (2008). Color filter incorporating a subwavelength patterned grating in poly silicon. Opt. Express.

[94] Chen C.M., Sung C.K. (2010). Fabricating metallic wire grating inside a polymeric substrate by insertion nanoimprint. Microelectron. Eng..

[95] Meng F., Luo G., Maximov I., Montelius L., Chu J., Xu H. (2011). Fabrication and characterization of bilayer metal wire-grid polarizer using nanoimprint lithography on flexible plastic substrate. Microelectron. Eng..

[96] Xue Y., Wang C., Zhang G., Cao B. (2011). Compound polarized wavelength filters with a single subwavelength structure. Optic. Commun..

[97] Yoon Y.T., Lee S.S., Lee B.S. (2012). Nano-patterned visible wavelength filter integrated with an image sensor exploiting a 90-nm CMOS process. Photon. Nanostruct..

[98] Tyo J.S., Goldstein D.L., Chenault D.B., Shaw J.A. (2006). Review of passive imaging polarimetry for remote sensing applications. Appl. Optic..

[99] Tyo J.S., Hayat M.M. (2007). Calibration and Compensation of Instrumental Errors in Imaging Polarimeters.

[100] Ratliff B.M., Tyo J.S., Black W.T., Boger J.K., Bowers D.L. (2008). Polarization visual enhancement technique for LWIR microgrid polarimeter imagery. Proc. SPIE.

[101] Bowers D.L., Boger J.K., Wellems L.D., Ortega S.E., Fetrow M.P., Hubbs J.E., Black W.T., Ratliff B.M., Tyo J.S. (2008). Unpolarized calibration and nonuniformity correction for long-wave infrared microgrid imaging polarimeters. Opt. Eng..

[102] Ratliff B.M., Tyo J.S., Boger J.K., Black W.T., Bowers D.L., Fetrow M.P. (2007). Dead pixel replacement in LWIR microgrid polarimeters. Opt. Express.

[103] Azzam R.M.A., Elminyawi I.M., El-Saba A.M. (1988). General analysis and optimization of the four-detector photopolarimeter. J. Opt. Soc. Am. A.

[104] Goldstein D.H., Chipman R.A. (1990). Error analysis of a Mueller matrix polarimeter. J. Opt. Soc. Am. A.

[105] Ratliff B.M., LaCasse C.F., Tyo J.S. (2009). Interpolation strategies for reducing IFOV artifacts in microgrid polarimeter imagery. Opt. Express.

[106] Gao S., Gruev V. (2011). Bilinear and bicubic interpolation methods for division of focal plane polarimeters. Opt. Express.

[107] Gao S., Gruev V. Gradient Based Interpolation for Division of Focal Plane Polarization Imaging Sensors.

[108] Xu X., Kullkarni M., Nehoirai A., Gruev V. (2012). A correlation-based interpolation algorithm for division-of-focal-plane polarization sensors. Proc. SPIE.

[109] Hecht E. (1998). Optics.

[110] Li M.M., Lu H.Q., Yin H., Huang X.L. Calibration and Error Analysis for Polarized-Light Navigation Sensor.

[111] Smith F.J. A New Algorithm for Navigation by Skylight Based on Insect Vision.

[112] Smith F.J. Insect Navigation by Polarized Light.

[113] Lu H., Huang X., Yin H. Principles and Applications of Polarized-Light-Aided Attitude Determination in Integrated Navigation.

[114] Hegedus R., Akesson S., Horvath G. (2007). Polarization patterns of thick clouds: Overcast skies have distribution of the angle of polarization similar to that of clear skies. J. Opt. Soc. Am. A.

[115] Hegedüs R., Bárta A., Bernáth B., Meyer-Rochow V.B., Horváth G. (2007). Imaging polarimetry of forest canopies: How the azimuth direction of the sun, occluded by vegetation, can be assessed from the polarization pattern of the sunlit foliage. Appl. Optic..

[116] Dacke M., Nilsson D.E., Warrant E.J., Blest A.D., Land M.F., O'Caroll D.C. (1999). Built-in polarizers form part of a compass organ in spiders. Nature.

[117] Barta A., Horvath G. (2004). Why is it advantageous for animals to detect celestial polarization in the ultraviolet? Skylight polarization under clouds and canopies is strongest in the UV. J. Theor. Biol..

[118] Seliger H.H., Lall A.B., Biggley W.H. (1994). Blue through UV polarization sensitivities in insects: Optimizations for the range of atmospheric polarization conditions. J. Comp. Physiol. A.

[119] Munz F.W., McFarland W.N., Crescitelli F. (1977). Evolutionary Adaptations of Fishes to the Photic Environment. Handbook of Sensory Physiology VII/5.

[120] Karman S.B., Diah S.Z.M., Futterknecht O., Gebeshuber I.C. Towards A “Navigational Sense” for Humans: Biomimetic Polarized Light Based Navigation System.

